# Gnathostomiasis: An Emerging Imported Disease

**DOI:** 10.3201/eid0906.020625

**Published:** 2003-06

**Authors:** David A.J. Moore, Janice McCrodden, Paron Dekumyoy, Peter L Chiodini

**Affiliations:** * Hospital for Tropical Diseases, London, U.K.; † Mahidol University, Bangkok, Thailand

**Keywords:** Gnathostoma, helminthiasis, imported disease, research

## Abstract

As the scope of international travel expands, an increasing number of travelers are coming into contact with helminthic parasites rarely seen outside the tropics. As a result, the occurrence of *Gnathostoma spinigerum* infection leading to the clinical syndrome gnathostomiasis is increasing. In areas where *Gnathostoma* is not endemic, few clinicians are familiar with this disease. To highlight this underdiagnosed parasitic infection, we describe a case series of patients with gnathostomiasis who were treated during a 12-month period at the Hospital for Tropical Diseases, London.

The ease of international travel in the 21st century has resulted in persons from Europe and other western countries traveling to distant areas of the world and returning with an increasing array of parasitic infections rarely seen in more temperate zones. One example is infection with *Gnathostoma spinigerum*, which is acquired by eating uncooked food infected with the larval third stage of the helminth; such foods typically include fish, shrimp, crab, crayfish, frog, or chicken. Previously, most disease related to *Gnathostoma* was reported from Southeast Asia, particularly Thailand and Japan, because of the dietary habits of those living there. In recent years, however, gnathostomiasis has become an increasing problem in Central and South America, most notably in Mexico (perhaps related to consumption of ceviche) (*1*,*2*). In cats and dogs, which serve as important reservoirs of infection in regions where *Gnathostoma* is endemic (*3*), the ingested third-stage larva matures into the adult worm in approximately 6 months (Figure 1). However, because the larva cannot mature into the adult form in humans, the third-stage larva can only wander within the body of the host; clinical symptoms of gnathostomiasis then occur because of the inflammatory reaction provoked by these migrating larvae (Figure 2).

**Figure 1:**
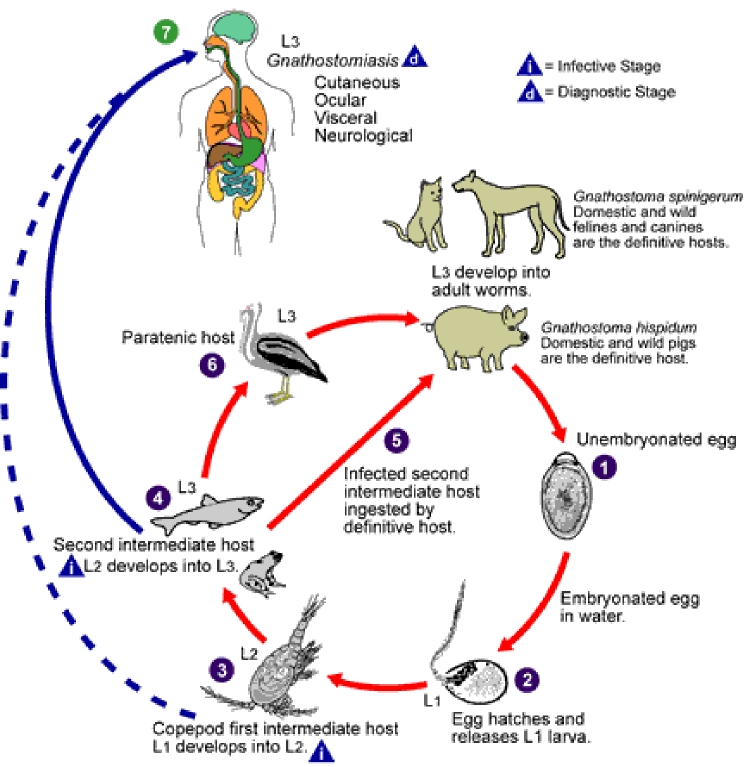
Life cycle of *Gnathostoma spinigerum*. Adapted from an original illustration by Sylvia Paz Diaz Camacho; available from: URL: http://www.dpd.cdc.gov/dpdx/HTML/gnathostomiasis.htm

**Figure 2:**
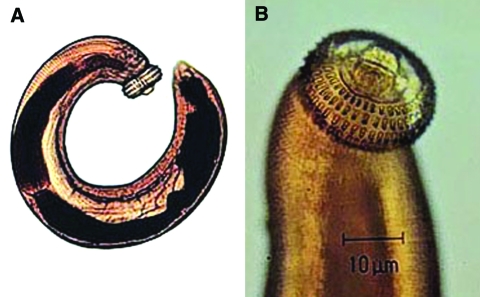
Third-stage larva of *Gnathostoma spinigerum*. A) whole larva; B) head. (Reproduced with the permission of Pichart Uparanukraw, Department of Parasitology, Faculty of Medicine, Chiang Mai University, Thailand.)

Traditionally the disease has been divided into cutaneous and visceral forms, depending on the site of larval migration and subsequent symptoms. Another form of gnathostomiasis, which is quite rare, includes the dangerous complication of central nervous system involvement (*4*). This form is manifested by painful radiculopathy, which can lead to paraplegia, sometimes following an acute (eosinophilic) meningitic illness.

We describe a series of patients in whom *G. spinigerum* infection was diagnosed at the Hospital for Tropical Diseases, London; they were treated over a 12-month period. Four illustrative case histories are described in detail. This case series represents a small proportion of gnathostomiasis patients receiving medical care in the United Kingdom, in whom this uncommon parasitic infection is mostly undiagnosed.

## Methods

The case notes of patients in whom gnathostomiasis was diagnosed at the Hospital for Tropical Diseases were reviewed retrospectively for clinical symptoms and confirmatory serologic results for the period April 1, 2000, to March 31, 2001. Clinical and laboratory data gleaned from case notes are described in the following sections.

### Definitions

The definition of clinical *Gnathostoma* infection is: 1) a history of intermittent, migratory skin and subcutaneous swellings (localized or not localized) with or without peripheral blood eosinophilia (eosinophil count >0.4 x 10^9^/L), or 2) otherwise undiagnosed eosinophilia with nonspecific symptoms. Plausible epidemiologic risk is defined as travel to an area in which gnathostomiasis had been reported previously (i.e., Southeast Asia and Central and South America). We did not impose a time limit on previous travel in our study. Positive *Gnathostoma* serologic results were defined as the presence on immunoblot of the specific 24-kDa band diagnostic of *Gnathostoma* infection (*5*,*6*). All serologic testing for gnathostomiasis was performed in the Department of Helminthology of the Faculty of Tropical Medicine at Mahidol University in Bangkok, Thailand. For patients at risk of *Loa loa* infection (because of previous travel to regions in central or West Africa where the infection is endemic), day-blood tests (samples taken between 12:00–2:00 p.m.) were performed to check for microfilaria and a filaria enzyme-linked immunosorbent assay was performed to exclude this diagnosis (Calabar swellings, indicative of *Loa loa* infection, may mimic gnathostomiasis).

## Results

During the 12-month study period, we identified 16 patients who had clinical symptoms consistent with *Gnathostoma* infection, a plausible epidemiologic risk, and positive serologic results. Seven patients were referred by their general practitioner (primary care physician) and four by consultant physicians working elsewhere in London. Median time from onset of symptoms to diagnosis was 12 months (range 3 weeks–5 years). A dietary history was recorded for three patients who reported eating (among other things) raw fish and watercress (patient 1); mutton, fish, and chicken in Bangladesh (patient 3); and fish and a variety of crustacea from market stalls in Southeast Asia (patient 13). Eosinophilia was noted in seven patients and was usually modest, always declining after treatment. Median erythrocyte sedimentation rate (available for 12 patients, data not shown) was 10 (range 1–62). The countries visited most frequently by our 16 patients were India (n=4), Bangladesh (n=3), China (n=2), and Thailand (n=2). Standard treatment during the period of study was albendazole (400 mg twice a day for 21 days). Three patients required a second course for recurrence of symptoms and incomplete resolution of eosinophilia.

### Case Histories

Detailed travel histories for these patients are described in the Table. The following sections include a case history for four patients; all of these patients had positive *Gnathostoma* serologic results and responded to albendazole therapy.

**Table:** Background information on patients in whom *Gnathostoma* infection was identified, April 1, 2000, to March 31, 2001, Hospital for Tropical Diseases, London^a^

Patient no.	Age	Referral source	Travel history	Eosinophil count (x 10^9^/L)^b^	Symptom duration
1^c^	26	GP	China, South Korea, Canada, Hong Kong, Tunisia	0.10	9 mo
2	26	General physician	Bangladesh, Italy	2.20	6 mo
3^c^	37	Rheumatologist	Bangladesh	4.37	3 y
4	28	HTD walk-in	Japan, Cuba	0.17	2 mo
5	35	GP	India, Sri Lanka	NA	3 y
6	34	HTD walk-in	South Africa, New Zealand, Jakarta, Singapore	0.80	3 mo
7	49	Dermatologist	India, Thailand	0.1	13 mo
8	51	GP	Sri Lanka, Brazil, Cambodia	0.08	2 y
9	26	Rheumatologist	India	1.33	3 y
10	27	GP	Bangladesh	1.10	5 y
11	23	GP	SE Asia, Australia	0.00	4 mo
12	25	self	Japan, SE Asia, USA, Canada	0.11	13 mo
13	24	HTD walk-in	SE Asia, India, China	0.96	3 wk
14^c^	49	Gastroenterologist	Far East, Caribbean, USA	0.95	12 mo
15	57	GP	Vietnam, Thailand	0.26	6 mo
16^c^	30	GP	Borneo, Belize, Ecuador, Peru, Australia	0.11	12 mo

^a^GP, general practitioner; HTD walk-in, Hospital for Tropical Diseases emergency walk-in clinic; NA, not available. ^b^Normal range 0–0.4 x 10^9^/l. ^c^Denotes case history in text.

### Case 1

A 26-year-old Asian woman, a resident of Hong Kong, was referred to our hospital by her primary care physician. She complained of the episodic appearance of “irritating” lumps on her limbs. Nine months earlier, the first of these lumps appeared on her right hand; since then, she had had a similar lump on her left foot and left hand, each lasting a few days and resolving spontaneously with no visible or palpable sequelae. Nine years previously, she had noted a lump rising near her left knee, which was followed 4 days later by a similar lump on her right thigh; both lumps had resolved spontaneously. All of the lumps were subcutaneous and estimated at 3–6 cm in diameter. A positive rheumatoid factor and anti-nuclear antibody >1:1,280 were noted. Her diet frequently included raw fish.

### Case 2

A 37-year-old woman from Bangladesh reported a 3-year history of intermittent swelling of the right forearm and upper arm to the midbiceps area associated with pruritus, myalgia, and arthralgia. The onset of her symptoms had occurred while she was visiting Bangladesh, where she had eaten mutton, fish, and chicken. An eosinophil count of 4.37 x 10^9^/L had prompted referral from a rheumatology clinic to the Hospital for Tropical Diseases. After a 21-day course of albendazole, her eosinophil count decreased to 1.12 x 10^9^/L; symptoms recurred several months later. After treatment with a second course of albendazole (400 mg twice a day for 21 days), her symptoms resolved and her eosinophil count returned to normal (0.25 x 109/L).

### Case 3

A 49-year-old Caucasian woman complained of a 12-month history of abdominal pain and symptoms suggesting gastroesophageal reflux. She had traveled widely in Southeast Asia 18 months earlier but denied eating crustacea or nonkosher meat. Gastric biopsy at upper gastrointestinal endoscopy demonstrated eosinophilic gastritis (peripheral blood eosinophil count of 0.95 x 10^9^/L), a finding that prompted serologic testing for *Gnathostoma*. Her symptoms resolved with albendazole treatment.

### Case 4

**Figure 3:**
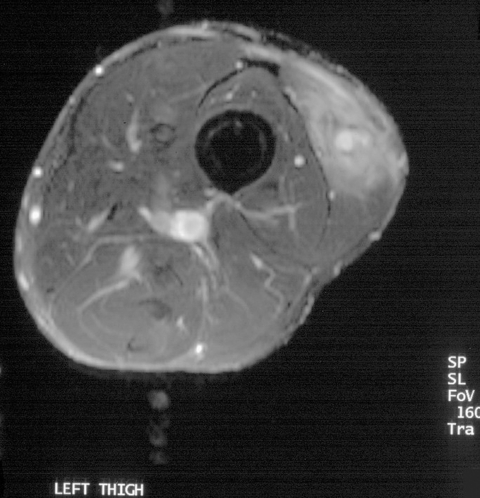
Magnetic resonance image of thigh with *Gnathostoma* larva (case 4).

Pain developed in the left thigh of a 30-year-old man while he was participating in the Eco-Challenge 2000 race in Borneo. A 4x3-cm lump in his thigh was initially attributed to a muscular tear; when this lump persisted for 12 months, he was referred to the Hospital for Tropical Diseases. Magnetic resonance imaging of the thigh (Figure 3) showed a lobulated lesion in the vastus lateralis muscle surrounded by edema. Serologic results were positive for *Gnathostoma*. Treatment with albendazole substantially reduced the size and firmness of the lesion but did not completely resolve it.

## Discussion

This series is the first reported set of travelers with gnathostomiasis. Patterns of international travel suggest that this condition may be seen more often in travelers and immigrants from regions in which the disease is endemic. Moreover, the widening geographic distribution of the infection and increasingly adventurous eating habits of visitors to such regions are likely to contribute to an increase in incidence.

In our patients, the median time from onset of symptoms to diagnosis was 12 months, which reflects both the intermittent, episodic nature of the symptoms and the obscurity of the diagnosis. We do not have data on the time to diagnosis after medical attention was sought, but anecdotally we often and understandably find a considerable delay.

The key to diagnosis of gnathostomiasis is recognition of the highly suggestive clinical history; cases 1 and 2 are the most typical. Once the disease is diagnosed, management is straightforward, but the rarity of the condition in areas in which the condition is not endemic might lead to the diagnosis’ being overlooked. The unusual symptoms, combined with the usual absence of physical signs between episodes, may lead to discounting of the symptoms and erroneous reassurance of the patient by clinicians unfamiliar with gnathostomiasis. Patients may be referred to rheumatology, dermatology, or general medical clinics; the absence of eosinophilia may also prevent due consideration of possible parasitic causes. Eosinophilia was present in only seven of our patients and thus cannot be considered as a screening tool. However, as a marker of treatment response in those with eosinophilia at baseline, this symptom was proven useful; for the three patients requiring a second course of albendazole, residual eosinophilia preceded symptom relapse.

Because of little information about dietary intake, we cannot comment on the sources of infection in our patients. More detailed dietary histories are now recorded routinely at the Hospital for Tropical Diseases, but the notorious inaccuracy of verbal dietary histories and the broad range of potential culprits eaten by many travelers suggest that, for identifying the source in humans, dietary history is usually of limited value.

A number of serologic tests are available for the diagnosis of gnathostomiasis. Our testing is performed at Mahidol University in Thailand by using an immunoblot to detect the specific 24-kDa band considered diagnostic of *Gnathostoma* infection. In that laboratory, for the four parasite-confirmed cases of *Gnathostoma*, the immunoblot was 100% sensitive, and antibodies of 15 parasitic diseases and one mixed infection were not cross-reactive, except for 1 of 13 samples from patients with paragonimiasis which gave a weak reaction against this antigen (*5*). Antibodies from 16 patients with confirmed cases of *Gnathostoma* were consistently reactive with this 24-kDa antigen. Cross-reactivity was not found in a further extensive study of parasitic and nonparasitic diseases (*6*).

The reported efficacy of albendazole in the treatment of gnathostomiasis is >90% (*7*,*8*), and similar success has been reported for ivermectin (*8*). Three of our patients required a second course of treatment. The episodic nature of this condition means that an initial determination is difficult as to whether cure has been effected, but the resolution of eosinophilia and lack of symptom recurrence within 12 months were taken as presumptive evidence of cure. Although we used a second course of albendazole for retreatment, ivermectin has also been used successfully (*9*).

A diagnosis of gnathostomiasis should be considered for patients with a history of transient, migratory cutaneous or subcutaneous swellings, or nonspecific gastrointestinal symptoms for which a potential epidemiologic exposure is identified. Management of the disease thereafter is usually relatively straightforward, although more than one course of treatment may be required to effect a cure.
